# Comparing nominal and real quality scores on next-generation sequencing genotype calls

**DOI:** 10.1186/1753-6561-5-S9-S14

**Published:** 2011-11-29

**Authors:** Alexander H Stram

**Affiliations:** 1 Zilkha Neurogenetic Institute, University of Southern California, 1501 San Pablo Street, Los Angeles, CA 90089, USA

## Abstract

I seek to comprehensively evaluate the quality of the Genetic Analysis Workshop 17 (GAW17) data set by examining the accuracy of its genotype calls, which were based on the pilot3 data of the 1000 Genomes Project. Taking advantage of the 1000 Genomes Project/HapMap sample intersect, I compared GAW17 genotype calls to HapMap III, release 2, genotype calls for an individual. These genotype calls should be concordant almost everywhere. Instead I found an astonishingly low 65.4% concordance. Regarding HapMap as the gold standard, I assume that this is a GAW17 data problem and seek to explain this discordance accordingly. I found that a large proportion of this discordance occurred outside targeted regions and that concordance could be improved to at least 94.6% by simply staying within targeted regions, which were sequenced across more samples. Furthermore, I found that in certain individuals, high sample counts did little to improve concordance and concluded that quality scores for a certain sample’s sequence reads were simply incorrect.

## Background

Although large-scale next-generation sequencing efforts such as the 1000 Genomes Project show promise in the further analysis and discovery of single-nucleotide polymorphisms (SNPs) and structural variants, there remain issues concerning the usability of these data. In my experience the data in the 1000 Genomes Project have not been particularly reliable. Take, for example, the case of rs76054577, a missense SNP on the *THADA* gene reported in the National Center for Biotechnology Information (NCBI) dbSNP database by the 1000 Genomes Project. A 16% minor allele frequency is reported in 25 Yoruba individuals [1] . Upon genotyping in my laboratory for further investigation, my co-workers and I found that this SNP was completely monomorphic in 269 African American individuals. Although the SNP has since been dropped from SNP lists in the 1000 Genomes Project, the 1000 Genomes Consortium reports that genotype accuracy rates can be as low as 70% [2], which alludes to the presence of many more “false” SNPs existing in 1000 Genomes Project data sets. This assertion prompted me to have reservations about the use of these data for detecting novel SNPs. The accuracy of the Genome Analysis Workshop 17 (GAW17) data set, which is based on the 1000 Genomes Project data, is therefore of particular interest. I seek to evaluate the accuracy of GAW17 genotype calls, which is an important consideration if these data are to be used as an exercise in discovering rare variants—a major discussion topic at GAW17.

## Methods

### Comparing GAW17 genotype calls to HapMap III

To assess the accuracy of GAW17 genotype calls, I must first establish a means to that end. I assume that HapMap genotype calls are reliable enough to constitute a de facto gold standard and are almost always correct. Because there is a sample overlap between HapMap III, release 2, and the 1000 Genomes Project, I can directly compare genotype calls from the 1000 Genomes Project to those from HapMap for calls made on the same loci for the same individuals. Specifically, I can compare GAW17 genotype calls to HapMap genotype calls: of the 697 individuals in the GAW17 data set, 616 are also in HapMap. Similarly, 3,403 of the 24,488 SNPs in the GAW17 data set are present in HapMap. Therefore there are 616(3,403) = 2,096,248 SNPs in the GAW17 data for which HapMap genotype calls are made on the same individual. By comparing these SNP genotype calls for each individual, I establish a concordance rate between GAW17 and HapMap genotype calls. Because I have assumed that HapMap is the gold standard data set that is most likely to represent the “true” genotype, I assume that discordant genotype calls are the fault of the GAW17 data set. If GAW17 genotype calls are sufficiently accurate, then their HapMap concordance rate should approach 100%.

### Generating quality scores for genotype calls

Genotype quality scores must be considered in any meaningful HapMap concordance analysis. Unfortunately, the GAW17 data set does not provide quality scores, and participants in the workshop were not told what quality filtering (if any) was done. Because it is unreasonable to expect high HapMap concordance for GAW17 genotype calls that may have been assigned low quality scores, I am forced to call my own genotypes for the 1000 Genomes data set, which GAW17 was based on. I then consider the quality scores given for each genotype call in the analysis. The analysis is concerned with the ability of the genotype quality scores to predict HapMap concordance rather than HapMap concordance itself.

Note that this analysis is not specific to the GAW17 data set and in fact bears direct relevance to the 1000 Genomes Project. The analysis is performed on genotype calls based on sequence data obtained directly from the 1000 Genomes Project, with the genotype calling done by means of software written specifically for the 1000 Genomes Project. If concordance problems with this new data set become apparent when quality scores are high, then we can view this as a general problem with the 1000 Genomes Project data, which could in turn be relevant to other large-scale next-generation sequencing projects.

I obtained the sequence alignment data for pilot3 study of the 1000 Genomes Project (July 2010 release). To simplify the analysis, I examine only chromosome 1 data for CEU individuals (northern and western European ancestry), which I hope is representative of the entire data set. Using the Broad Institute’s Genotype Analysis Toolkit (GATK) software, I make genotype calls based on the sequence data provided for these 90 CEU samples, 84 of which are in HapMap. For each genotype call made by GATK, we are given a quality score, which is a Bayesian function of relevant sequence reads, sequence read quality scores, and sequence read mapping quality scores. If these quality scores are accurate, then HapMap concordance should reflect this accordingly: for *n* genotypes called with (1 – *p*) confidence, we expect that *n*(1 – *p*) genotypes are HapMap concordant.

## Results

### Comparing GAW17 genotype calls to HapMap III

Of the 2,095,632 genotype calls in the GAW17 data set for which HapMap comparison can be done, a mere 1,371,479, or 65.4%, of genotype calls are HapMap concordant. The most concordant individual is 73.63% concordant; the lowest is 51.79% concordant. To put this in perspective, consider that the average HapMap minor allele frequency of these 3,402 SNPs is 20.96%. Assuming Hardy-Weinberg equilibrium, one can attain 62.47% HapMap concordance by simply guessing that every genotype is major-allele homozygous. This issue clearly warrants further inquiry.

### Generating quality scores for genotype calls

A total of 279,491 genotypes are called on chromosome 1 for CEU individuals in HapMap for which the SNP is also in HapMap. Looking only at the 44,650 calls made with 99% confidence or greater, I find 39,506/44,650 = 88.48% overall HapMap concordance. This immediately points to the quality scores being inaccurate. Assuming that each genotype quality score is independent and truly called with 99%+ accuracy, we can calculate the probability of seeing 88.48% or less concordance on 44,650 genotypes using the binomial distribution:

*P*(≤ 39,506 successes in 44,650 trials | *P* of success ≥ 99%) < 2.23 × 10^–308^. (1)

By examining the concordance rate for each sample, I find that, using the binomial distribution, 30 of 84 samples have concordance rates that do not correspond to their quality scores (*α* = 10^–4^). For example, individual NA12748 has 54.66% concordance on 2,534 calls, NA12842 has 46.15% concordance on 1,703 calls, and NA12889 has 42.10% concordance on 1,240 calls, with all calls having a quality score of 99%+. If the GAW17 data set were filtered only on these inaccurate quality scores, then the resulting HapMap discordance would seem inevitable.

## Discussion

The prime suspect in these discordance issues is inaccurate prior probabilities. Not every SNP is sequenced on every individual, resulting in varying sample counts across SNPs. Because Bayesian genotype callers such as GATK are conditioned on a multisample prior probability (i.e., allele frequencies are estimated using all available samples), a low sample count in this prior probability estimate would yield high variance in the estimate. If this variance is large and not accounted for in confidence scores, then inaccuracy would certainly result. GATK’s inaccurate quality scores appear to be at least partly explained by such variance: For those loci for which 10 or more samples were included in the prior probability calculation, we see 95.13% concordance versus 54.62% for areas with fewer than 10 samples. However, considering variance in prior probabilities does not solve all the data’s problems. In fact, this may only be reducing the effect of bad samples.

A significant number of samples have pathological concordance rates, even when considering only high-sample genotype calls. Although most showed improvement in concordance when only those genotype calls over 10 or more individuals were considered, 12 samples did not have concordance rates possible under their supposed 99%+ confidence scores (*α* = 10^–4^). The most extreme case is sample NA12829, which sees 51.15% concordance for 520 genotype calls, each made with 99%+ confidence and over 10 or more samples, with no improvement even on those genotypes called over all individuals in the CEU sample. To completely eliminate the poor prior possibility on samples such as NA12829, I did my own Bayesian calculations using the sequence read and alignment quality scores given for the respective individual’s alignment files, conditioning with prior probabilities that were based on the HapMap allele frequencies. HapMap concordance was still far too low. Having apparently exhausted all other explanations, I am forced to conclude that for certain samples, the base quality scores given by the 1000 Genomes Project data are overstated.

## Conclusions

Although I have focused the analysis on known SNPs (particularly those in HapMap), discordance on known SNPs, which points to inaccurate genotype calls, will likely point to inaccurate genotype calls on novel SNPs, making the discovery of rare variants a much more difficult task. The concordance of the GAW17 data set with HapMap can be improved to at least 96.4% simply by staying within targeted regions, which are high-sample areas by definition. I imagine that this could be improved on further if the imputation process was limited to these regions, thus completely eliminating the effect of data from untargeted regions. However, it appears that a significant number of samples in the GAW17 data set have incorrect genotype calls and therefore incorrect quality scores, and, whether this is due to sample contamination, sample mix-up, or poor sequencing, these samples should be excluded from analysis altogether. Finally, I recognize that the 1000 Genomes Project is a work in progress, and data quality assessment will undoubtedly improve in future releases.

## Competing interests

The author declares that there are no competing interests.

## Authors’ contributions

AHS carried out all the work.
